# Presence of adipose tissue along the posteromedial tibial border

**DOI:** 10.1186/s40634-021-00408-0

**Published:** 2021-10-20

**Authors:** Okunuki Takumi, Tanaka Hirofumi, Akuzawa Hiroshi, Yabiku Hiroki, Maemichi Toshihiro, Matsumoto Masatomo, Hoshiba Takuma, Kumai Tsukasa

**Affiliations:** 1 Graduate School of Sport Sciences, Waseda University, Saitama, Japan; 2 Hyakutake Orthopedic & Sports Clinic, Saga, Japan; 3 Faculty of Sport Sciences, Waseda University, Saitama, Japan; 4 Department of Orthopedic Surgery, University of Ryukyus, Okinawa, Japan; 5 Kuwana City Medical Center, Kuwana, Mie Japan; 6 Waseda Institute for Sport Sciences, Saitama, Japan

**Keywords:** Adipose tissue, Gross anatomy, MRI, Ultrasound, Fat quantification, Imaging

## Abstract

**Purpose:**

The flexor digitorum longus and posterior tibial tendon as well as the perforating veins are located along the distal posteromedial tibial border. Adipose tissue may surround these structures and possibly play a role in reducing mechanical stress. This study aimed to examine the adipose tissue along the posteromedial tibial border via magnetic resonance imaging (MRI), ultrasound, and gross anatomical examination.

**Methods:**

The lower legs of 11 healthy individuals were examined every 3 cm from the medial malleolus using MRI and ultrasound. The fat fraction was calculated using fat fraction images. In addition, the gross anatomy of the flexor digitorum longus origin and adipose tissue along the posteromedial tibial border was examined in seven fresh cadavers. The fat fraction was compared at different heights along the posteromedial tibial border and in Kager’s fat pads; we also compared the height of the flexor digitorum longus origin and adipose tissue.

**Results:**

In vivo, the adipose tissue was identified along the entire posteromedial tibial border using MRI and ultrasound. There was no significant difference in fat fraction between Kager’s fat pads and the adipose tissue along the posteromedial tibial border, except at the 6 cm mark. All seven cadavers presented adipose tissue along the posteromedial tibial border, significantly more distal than the flexor digitorum longus origin.

**Conclusion:**

The adipose tissue was identified along the posteromedial tibial border via MRI, ultrasound, and gross anatomical examination; thus, this tissue may play a role in reducing friction and compressive stress in tendons.

## Introduction

The adipose tissue exists around vessels and tendon attachments and plays a role in reducing friction and compressive stress [1, 3, 13]. However, excessive friction and compressive stress lead to adipose tissue inflammation in the ankle and knee joints [4, 21]. These findings indicate that the adipose tissue causes pain. Therefore, it is important to determine the adipose tissue distribution for treatment in the field of orthopaedics.

Along the distal posteromedial tibial border, the flexor digitorum longus (FDL) and posterior tibial (PT) tendon run with many perforating veins running perpendicular to the long axis of the distal tibia [2, 5, 14]. The adipose tissue would be present along the posteromedial tibial border to decrease friction or compressive stress. Furthermore, approximately 30%–60% of patients with medial tibial stress syndrome complain of pain along the distal posteromedial tibial border (0%–33%) [17, 23]. Therefore, the adipose tissue along the posteromedial tibial border could be a symptom site and cause medial tibial stress syndrome; however, whether the adipose tissue is present in this area has not yet been elucidated.

Two- and six-point Dixon gradient-echo magnetic resonance imaging (MRI) have been shown to provide stable water–fat signal separation for dual-echo imaging using phase information to resolve water–fat ambiguity in chemical-shift imaging [8, 22]. Previous studies used these imaging protocols to investigate the fat fraction in the gastrocnemius muscle and rotator cuff and showed that signal intensity correlates with fat fraction [8, 22]. Thus, we hypothesised that MRI could be used to evaluate whether the adipose tissue exists along the distal posteromedial tibial border. Additionally, the pathology of the adipose tissue in the knee, ankle, and heel was evaluated using ultrasound [4, 12, 21]. Ultrasound can also be used to evaluate the adipose tissue along the distal posteromedial tibial border. Thus, this study aimed to investigate the adipose tissue along the posteromedial tibial border using MRI and ultrasound. After MRI and ultrasound evaluations, we confirmed the presence of the adipose tissue via gross anatomical examination.

## Materials and methods

### In vivo study

#### Participants

Eleven healthy individuals participated in this study (men, *n* = 5; women, *n* = 6; age, 22.9 ± 1.2 years; height, 165.8 ± 7.3 cm; weight, 58.8 ± 11.6 kg). None of the participants had complaints of injuries or pain in their legs. Their right lower legs were examined using MRI and ultrasound. This study was approved by our Institutional Ethics Review Board (2020–139), and written informed consent was obtained from all participants.

#### MRI examination

**Fig. 1:**
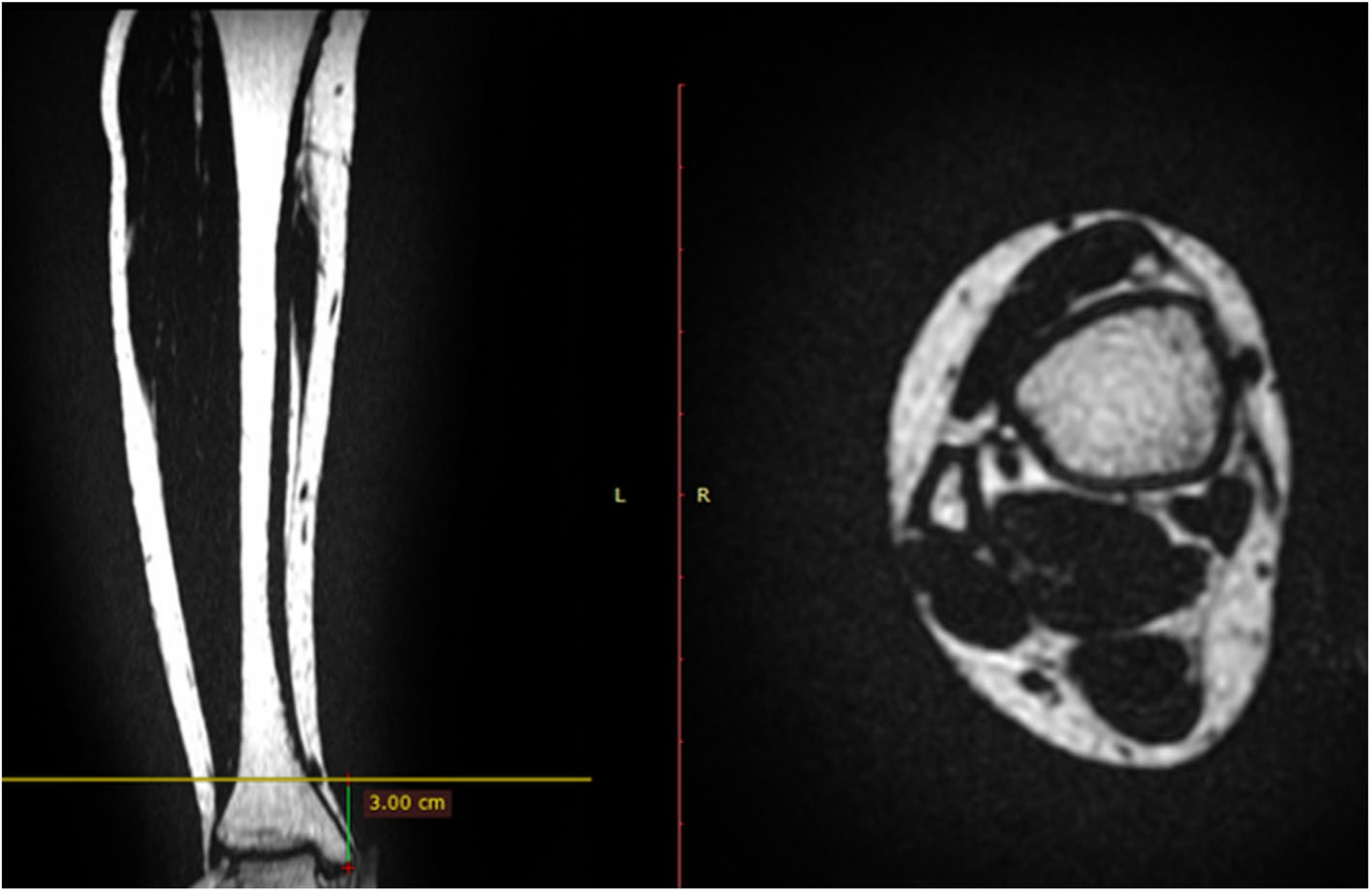
Fat-only images using LAVA-flex in a 22-year-old woman. Left: Coronal plane image reconfigured from transverse images. The vertical line from the medial malleolus was drawn by the author. The transverse line in the coronal plane image was drawn based on the selected transverse plane. Right: Transverse plane image at 3 cm from the medial malleolus. Transverse plane images were obtained during the experimental protocol

The MRI examinations were performed using a 3.0-T scanner (SIGNA Premier; GE Healthcare, Chicago, IL, USA). Participants laid in the supine position with their ankles in the neutral position. LAVA-Flex and Ideal IQ images were obtained in the transverse plane using two- and six-point Dixon gradient-echo MRI, respectively. We obtained fat-only, water-only, and in-phase images using LAVA-Flex, and fat fraction images using Ideal IQ. In-phase imaging indicates T1-weighted imaging. In addition, T2-weighted imaging and short-tau inversion recovery (STIR) images were obtained in the transverse images. We extracted transverse plane images every 3 cm from the distal tip of the medial malleolus up to 27 cm. To measure the distance from the medial malleolus, coronal plane images were reconfigured from the fat-only transverse plane images using imaging analysis software (RadiAnt DICOM Viewer; RadiAnt, Poznań, Poland) (Fig. 1). Coronal and transverse images were correlated. The distance from the distal tips of the medial malleolus was calculated based on the number of slices. The soft tissue enclosing the adipose tissue was also identified. LAVA-Flex can accurately separate fat and water by utilising the difference in chemical shifts between protons in water and fat molecules [22]. Signals from fat are suppressed on water-only imaging, whereas signals from water are suppressed on fat-only imaging. Thus, the adipose tissue was defined based on high signal intensity on fat-only imaging as well as low signal intensity on water-only imaging. Additionally, the soft tissue enclosing the adipose tissue was identified using in-phase and water-only images [8, 22].

##### Quantitative evaluation of the fat fraction

Fat fractions of the adipose tissue, which were defined on MRI, along the posteromedial tibial border and Kager’s fat pads were calculated using fat fraction images. As Kager’s fat pad is a known type of adipose tissue, it served as a basis for identifying the adipose tissue along the posteromedial tibial border. Fat fraction images were extracted every 3 cm from the distal tip of the medial malleolus up to 27 cm and 1–2 cm from the distal tip of the medial malleolus; the fat fractions of the adipose tissue along the posteromedial tibial border and Kager’s fat pad were then quantified. After the images were extracted, the image datasets were imported into MATLAB (Rev. 2020a; The MathWorks, Natick, MA). The fat fraction was calculated from signal values within the range of interest; these were created for all images. The range of interest was created by author T.O. The intraclass correlation coefficient values were 0.865–0.982 for the fat fraction at each height of the posteromedial tibial border and for Kager’s fat pads.

#### Ultrasound

Ultrasound examinations were performed by a physiotherapist (8 years of clinical experience) using a Toshiba Aplio scanning machine (Canon Medical Systems Corporation, Tochigi, Japan). Participants sat with their knees flexed at 90° and their ankles in the neutral position. Their lower legs were marked every 3 cm from the distal tip of the medial malleolus, and high-frequency (5–14 MHz) linear-array transducers (length: 58 mm) were transversely placed along the posteromedial tibial border. We identified and recorded the tissue that appeared to be the adipose tissue.

### Cadaver study

#### Materials

A total of seven fresh-frozen human cadaver feet and lower legs were obtained from a tissue bank. The proximal tibia was already cut, and we excised the skin longitudinally and transversely along the medial lower leg and medial malleolus, respectively. Subcutaneous fat was removed from the crural fascia and tibia. The crural fascia was excised longitudinally and transversely from the medial gastrocnemius muscles and medial malleolus, respectively. The very thin fascia of the deep posterior compartment was identified; this could be easily peeled off to confirm the presence of the FDL and SOL originating from the posteromedial tibial border. After incision of the SOL origin, we observed that the FDL originated from the posteromedial tibial border. Two orthopaedic clinicians (each with > 20 years of clinical experience) evaluated the presence of the adipose tissue along the posteromedial tibial border. The distance from the most distal end of the FDL origin and the most distal adipose tissue to the distal tips of the medial malleolus was measured using a tape measure.

### Statistical analysis

For the in vivo study, the presence of adipose tissue and surrounding soft tissue on MRI was determined every 3 cm from the distal tip of the medial malleolus. The Shapiro–Wilk test revealed that the fat fraction was normally distributed along the posteromedial tibial border. The fat fractions between different heights along the posteromedial tibial border and Kager’s fat pad were compared using repeated-measures analysis of variance. Subsequently, a paired *t*-test was performed for paired comparisons. In the cadaver study, the Shapiro–Wilk test revealed that the distance of the distal ends of the FDL origin and that of the distal ends of adipose tissue were normally distributed. The distance from the distal ends of the FDL origin was compared to that of the distal ends of adipose tissue using a paired *t-*test. The significance level was set at 0.05. Statistical analyses were performed using IBM SPSS version 27 (SPSS Inc., Chicago, IL, USA).

## Results

### In vivo study

#### MRI

**Fig. 2:**
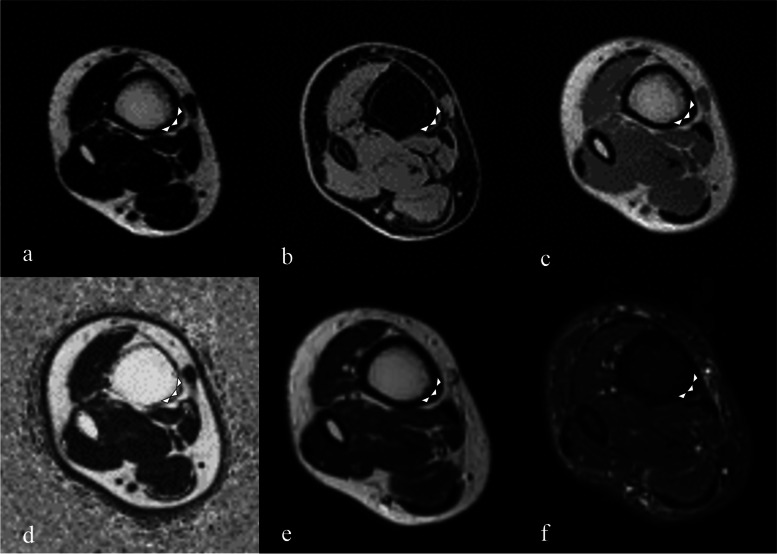
A 23-year-old man. Images in transverse plane were acquired 6 cm from the distal tip of the medial malleolus. **a**: Fat-only image obtained using LAVA-Flex. **b**: Water-only image obtained using LAVA-Flex. **c**: In-phase image (same T1-weighted imaging) obtained using LAVA-Flex. **d**: Fat fraction image obtained using Ideal IQ. **e**: T2-weighted image. **f**: short-tau inverse recovery (STIR). Adipose tissue positioned on the posteromedial tibial border (white arrowheads). The adipose tissue shows high signal intensity on fat-only, in-phase, fat fraction, and T2-weighted images. On the other hand, the adipose tissue shows low signal intensity on water-only and STIR images

**Fig. 3:**
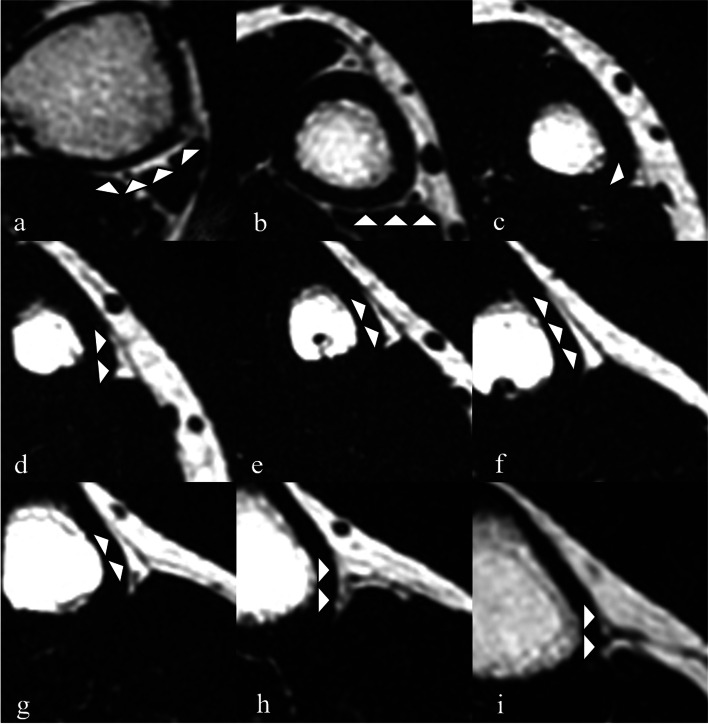
A 22-year-old man. Adipose tissue morphology by slice levels on fat-only transverse plane images using LAVA-Flex. Images acquired at **a**: 3 cm; **b**: 6 cm; **c**: 9 cm; **d**: 12 cm; **e**: 15 cm; **f**: 18 cm; **g**: 21 cm; **h**: 24 cm; and **i**: 27 cm from the distal tip of the medial malleolus. These images are from the same participant as in Fig. 6

**Fig. 4:**
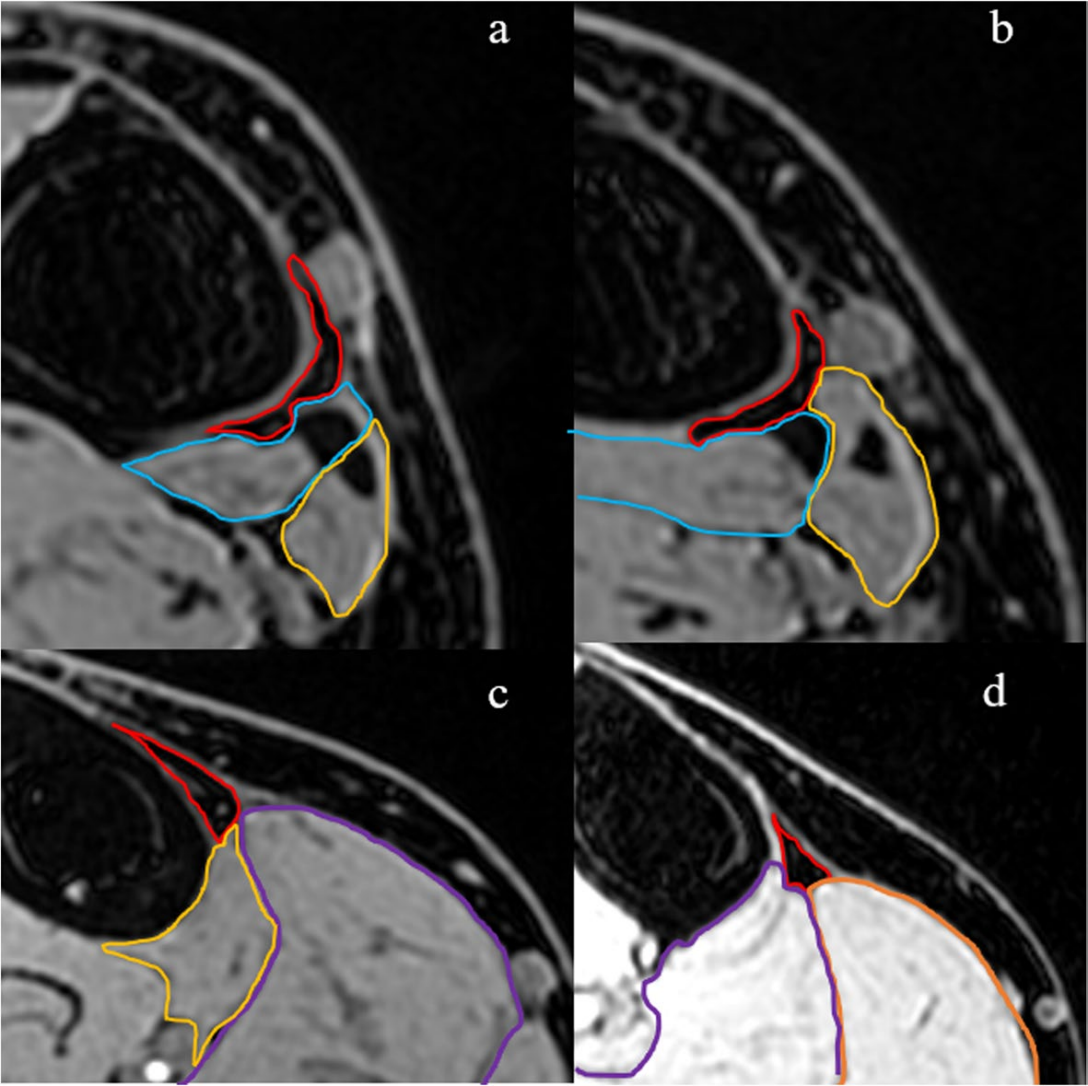
**a, b**, and **c**: A 23-year-old man. **d**: A 24-year-old man. Water-only transverse plane images obtained using LAVA-Flex show soft tissue enclosing the posterior aspect of the adipose tissue. Images acquired at **a**: 6 cm; **b**: 9 cm; **c**: 21 cm; and **d**: 27 cm from the distal tip of the medial malleolus. Red line: adipose tissue; blue line: posterior tibialis muscle and tendon; yellow line: flexor digitorum longus muscle and tendon; purple line: soleus muscle; orange line: medial head of the gastrocnemius

In vivo, tissue that appeared to be the adipose tissue was identified along the posteromedial tibial border and soft tissue that enclosed the adipose tissue in all participants. The tissue had low signal intensity on water-only images, whereas the same tissue had high signal intensity on fat-only, in-phase (same as T1-weighted), T2-weighted, and fat fraction images (Fig. 2); its morphology was triangular. On STIR, the tissue could not be identified due to low intensity (Fig. 2). We identified this tissue to be the adipose tissue. The adipose tissue was present in all participants at a distance of 6–24 cm proximal to the medial malleolus. Eight participants had the adipose tissue at the 3 cm mark, whereas three had the adipose tissue present at the 27 cm mark (Fig. 3). The adipose tissue was enclosed by the posteromedial tibial border, crural fascia, and tendon or muscle of the lower leg (Fig. 4). The PT muscle and tendon were positioned on the posterior side of the tissue at the 3 and 6 cm marks, respectively, in all participants (Fig. 4a). In one participant, the PT tendon was positioned at the 9 cm mark. Furthermore, the FDL muscle belly enclosed the posterior side of the adipose tissue from the 6 to 21 cm marks (Fig. 4b). The soleus muscle belly enclosed the posterior side of the adipose tissue from the 15 to 27 cm marks (Fig. 4c). The medial head of the gastrocnemius muscle enclosed the adipose tissue from the 24 to 27 cm marks (Fig. 4d). Some portions of the PT tendon and FDL muscle belly, FDL muscle belly and soleus, and soleus and medial gastrocnemius overlapped.

##### Quantitative measure of the fat fraction

**Fig. 5:**
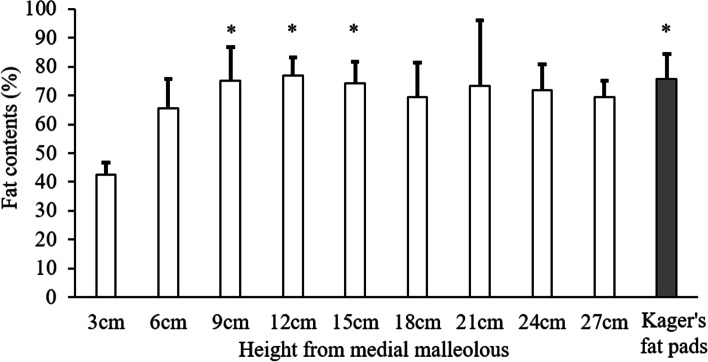
Fat fraction of the adipose tissue at each height along the posteromedial tibial border and that of Kager’s fat pads. The fat fraction at 6 cm is significantly lower than that at the 9, 12, 15 cm marks and that of Kager’s fat pads (*P* < 0.05). The fat fractions at 3 and 27 cm represent measurements from eight and four participants, respectively. *: significant difference compared with the fat fraction at the 6 cm mark

The fat fraction of Kager’s fat pad was 75.8% ± 8.8% and that of the adipose tissue along the posteromedial tibial border was 42.5%–77.0% (Fig. 5). We only compared the fat fraction of the adipose tissue along the posteromedial tibial border from the 6 to 24 cm marks with that of Kager’s fat pads because these were confirmed to be present in 11 participants. There was no significant difference between the fat fraction of the adipose tissue along the posteromedial tibial border from the 9 to 24 cm marks and that of Kager’s fat pad. However, the former had a significantly lower fat fraction at the 6 cm mark than that at the 9, 12, and 15 cm marks as well as than the fat fraction of Kager’s fat pad (Fig. 5).

#### Ultrasound

**Fig. 6:**
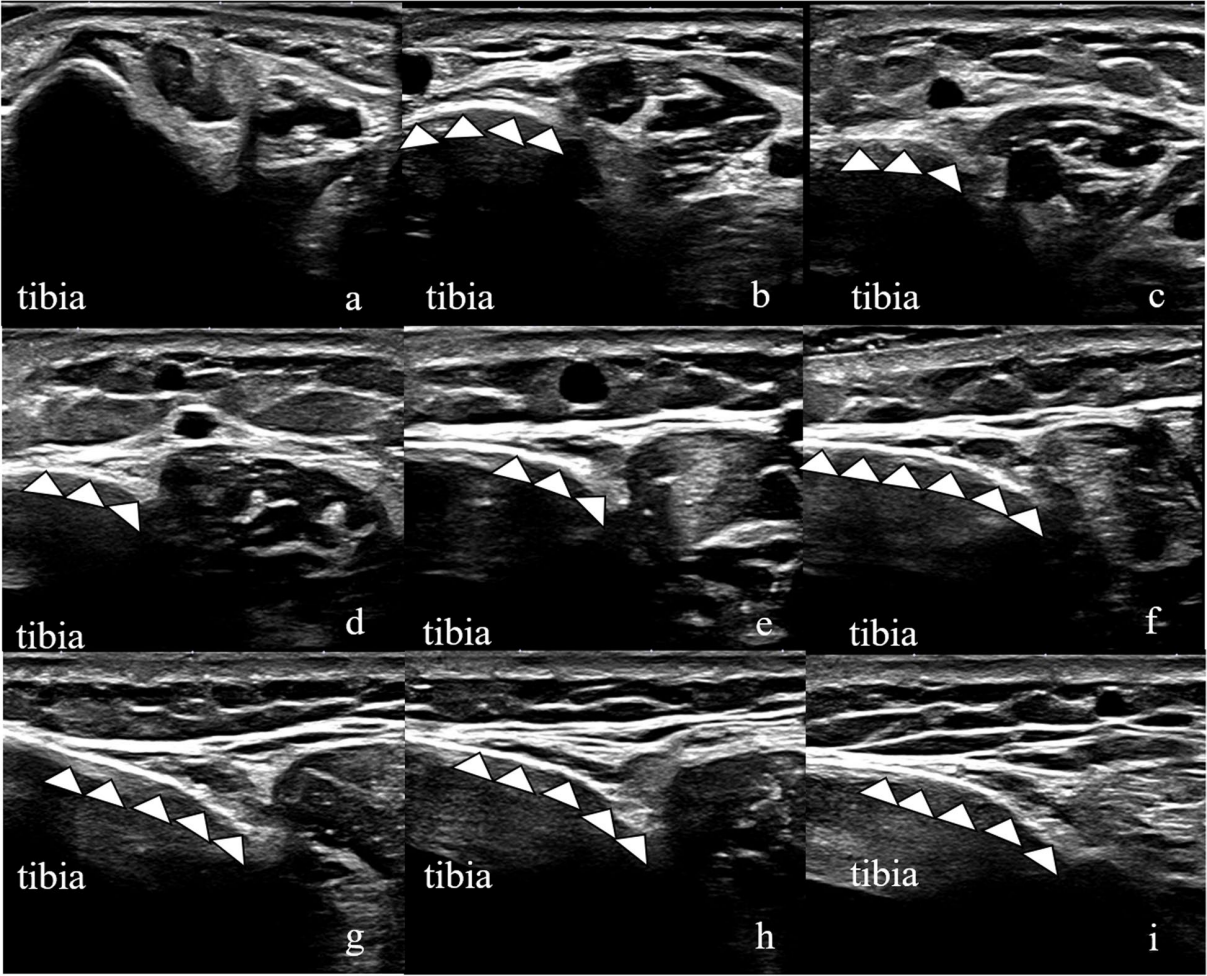
A 22-year-old man. Ultrasound images of the transverse axis of the medial calf. The adipose tissue is visualised with high echogenicity (white arrows). Images acquired at **a**: 3 cm; **b**: 6 cm; **c**: 9 cm; **d**: 12 cm; **e**: 15 cm; **f**: 18 cm; **g**: 21 cm; **h**: 24 cm; and **i**: 27 cm from the distal tip of the medial malleolus. These images are from the same participant as in Fig. 4

The adipose tissue could be identified using ultrasound and was observed as a hyperechoic region along the posteromedial tibial border (Fig. 6). Its morphology on ultrasound images was similar to that on MRI at the same height (Fig. 6a–h).

### Cadaver study

**Fig. 7:**
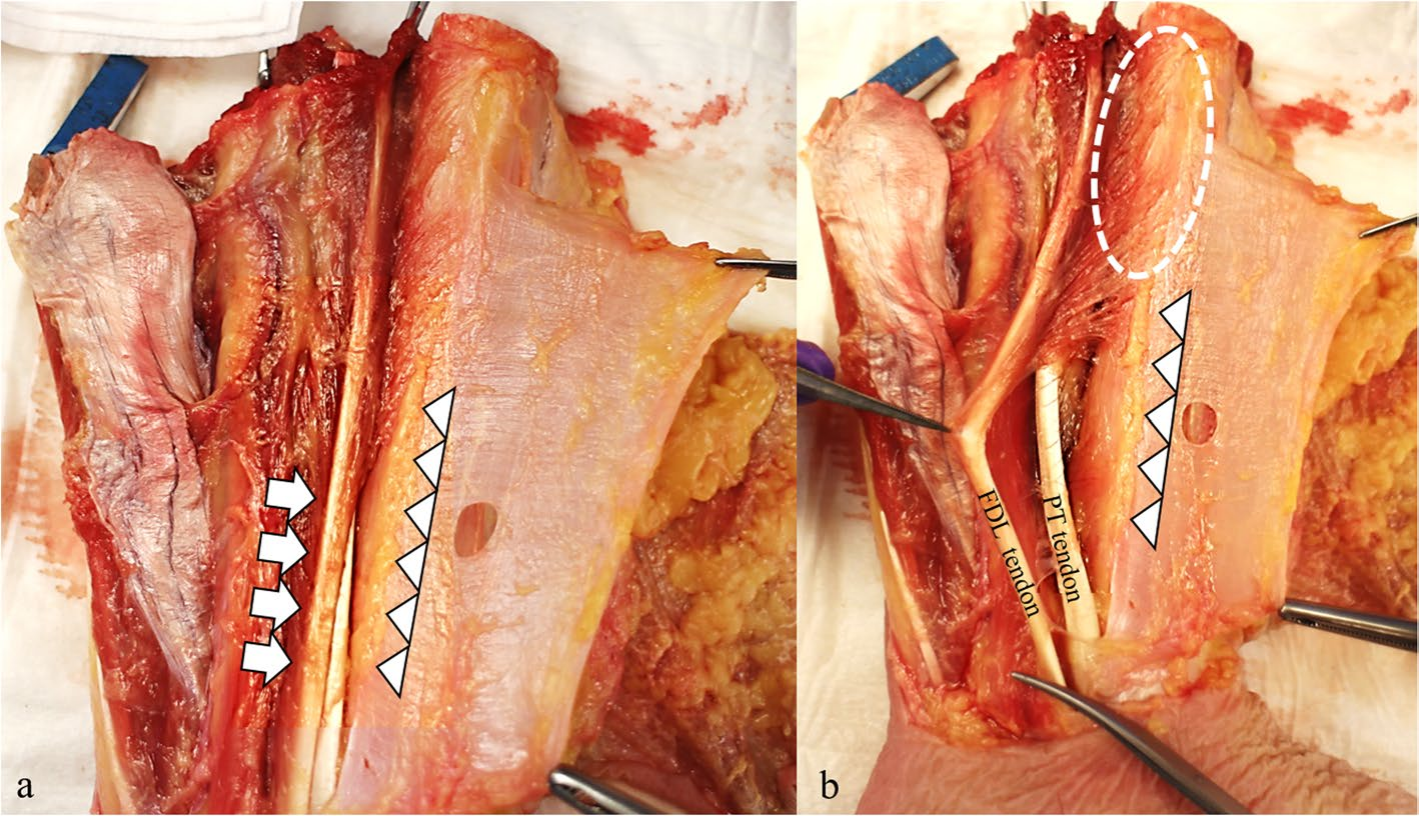
The crural fascia is excised and opened on the left lower leg. The adipose tissue (yellow soft tissue) is confirmed along the posteromedial tibial border (white arrowhead). **a**: The adipose tissue is present in front of the flexor digitorum longus (FDL) muscle–tendon and posterior tibialis muscle (PT) tendon (white arrow). **b**: Pulled FDL origin (white dashed line circle) seen at the more proximal adipose tissue

The FDL muscle originated from the posteromedial tibial border in all cadavers. The adipose tissue was observed (Fig. 7a and b); it was located between the FDL or PT tendon and the posteromedial tibial border (Fig. 7a). The most distal measurements of the FDL origin and adipose tissue were 8.1 ± 1.1 and 5.8 ± 2.1 cm, respectively. A significant difference in the distance from the medial malleolus between the FDL origin and adipose tissue was noted (*P* = 0.017).

## Discussion

The purpose of this study was to investigate the presence of the adipose tissue along the posteromedial tibial border on MRI and ultrasound in vivo and to confirm the presence of the adipose tissue based on gross anatomical evaluation. The current study revealed the presence of the adipose tissue along the posteromedial tibial border on MRI and ultrasound; the tissue was positioned more distally than the FDL origin according to gross anatomical evaluation. Thus, these results supported our hypothesis.

Fat-only, water-only images (LAVA-Flex), and fat fraction images (Ideal IQ) were used to identify the adipose tissue along the posteromedial tibial border. LAVA-Flex and Ideal IQ were previously used to evaluate the fat fraction of the rotator cuff [22]. The adipose tissue showed low signal intensity on water-only images and high signal intensity on fat-only images [22]. Therefore, we defined tissue with low signal intensity on water-only images and high signal intensity on fat-only images as the adipose tissue (Fig. 2). The fat fraction at the 9–24 cm marks was not significantly different from that of Kager’s fat pads (Fig. 5). These results suggest that the adipose tissue at the 9–24 cm marks, which we defined, contains as much fat as Kager’s fat pads. On gross anatomical examination, we confirmed the presence of the adipose tissue along the posteromedial tibial border (Fig. 7). These tissues were identified using the general MRI protocol: high signal intensity on T1-weighted (same in-phase images in this study) and T2-weighted images and low signal intensity on STIR images. Ultrasound showed high echogenicity along the posteromedial tibial border, and the morphology was similar to that on MRI (Fig. 6). These results indicate that we positively identified the adipose tissue along the posteromedial tibial border on T1-weighted, T2-weighted, and ultrasound images.

The adipose tissue was more distal than the FDL origin and was positioned between the PT or FDL tendon and the tibia on gross anatomical examination (Fig. 7). The adipose tissue between tendons and bones plays a role in reducing the mechanical stress acting upon tendons [1, 3]. Therefore, the adipose tissue along the distal posteromedial tibial border possibly plays a role in reducing mechanical stress. Previous studies reported that excessive mechanical stress causes adipose tissue inflammation in the fat pad region at the ankle and knee joints [20, 21]. In a histological study, inflammation would induce infiltrate angiogenesis and increase fibrosis in the adipose tissue [7, 10]. Ultrasound showed vascularisation and increased echogenicity for the inflamed heel fat pad and Kager’s fat pad, respectively [6, 11, 21]. High muscle echogenicity on ultrasound indicates that vessels and fibrosis were abundant [18]. In this study, the adipose tissue showed high echogenicity on ultrasound images. These reasons may indicate that the adipose tissue along the posteromedial tibial border was inflamed due to excessive mechanical stress and has abundant vessels and fibrosis. The adipose tissue at the 6 cm mark, which had a lower fat fraction, is possibly the result of inflammation caused by exposure to excessive mechanical stress by muscle tendons. These findings indicate that the adipose tissue along the distal posteromedial tibial border possibly plays a role in reducing friction and compressive stress.

A study by Moses et al. reported that perforating veins are abundant along the posteromedial tibial border [14]. These veins are most abundant 7–13 cm and 23–27 cm proximal to the medial malleolus [14], where the presence of the adipose tissue was confirmed in this study. These veins run along different nerves [15, 16, 19]; in particular, the small saphenous vein is often located close to the cutaneous nerve of the calf, which pierces the crural fascia [15]. In addition, the adipose tissue around the tendon attachments contains nerve endings and veins, according to histological studies [1]. Thus, the adipose tissue and perforating veins are closely related to the nerves, suggesting that the adipose tissue is the source of pain along the posteromedial tibial border.

In patients with heel or knee pain, T1- and T2-weighted imaging demonstrated reduced signalling in the heel fat pad and Hoffa’s fat pad [4, 6]. In patients with spondyloarthropathy, the subcutaneous lumbar fat appeared to have high signal intensity on STIR images [9]. These findings indicate that we could evaluate adipose tissue abnormalities in patients using the general MRI protocol that we used. On ultrasound, the heel fat pad and Kager’s fat pad in patients with heel pain and Achilles tendinopathy had increased vascularity, thickness, and echogenicity [6, 11, 21]. We may confirm these adipose tissue abnormalities along the posteromedial tibial border via ultrasound. In the future, the adipose tissue along the posteromedial tibial border on T1-weighted (same in-phase imaging), T2-weighted, and STIR imaging and ultrasound in patients with medial shin pain, including those with medial tibial stress syndrome, should be evaluated.

This study has some limitations. The amount of nerve and fibrous tissue in the adipose tissue along the posteromedial tibial border remains unclear. Therefore, whether the adipose tissue has nerves and fibrous tissues along the distal posteromedial border should be investigated in a histological study. Further, it is unclear whether mechanical stress, such as friction and compression, could be generated on the adipose tissue. In the future, the relationship between mechanical stress and adipose tissue must be further explored.

In conclusion, this study revealed the existence of adipose tissue along the posteromedial tibial border. The adipose tissue may play a role in reducing friction and compressive stress caused by the FDL and TP tendons. As the adipose tissue can be identified on MRI and ultrasound, these imaging techniques may be used to evaluate symptoms in patients with pain along the posteromedial tibial border.

## Data Availability

The datasets used and/or analysed in the current study are available from the corresponding author on reasonable request.

## Abbreviations

MRI: Magnetic resonance imaging
STIR: Short-tau inversion recovery
FDL: Flexor digitorum longus
PT: Posterior tibial
